# Distribution of plastids and mitochondria during male gametophyte formation in *Tinantia erecta* (Jacq.) Fenzl

**DOI:** 10.1007/s00709-019-01363-5

**Published:** 2019-03-09

**Authors:** Rafał Marciniec, Emil Zięba, Krystyna Winiarczyk

**Affiliations:** 1 Department of Plant Anatomy and Cytology, Maria Curie-Skłodowska University, Akademicka 19, 20-033 Lublin, Poland; 2 Confocal and Electron Microscopy Laboratory, Centre for Interdisciplinary Research, John Paul II Catholic University of Lublin, Al. Kraśnicka 102, 20-718 Lublin, Poland

**Keywords:** Microsporogenesis, Microgametogenesis, Chondriokinesis, Plastids, Mitochondria, *Tinantia erecta*

## Abstract

During meiosis in microsporogenesis, autonomous cellular organelles, i.e., plastids and mitochondria, move and separate into daughter cells according to a specific pattern. This process called chondriokinesis is characteristic for a given plant species. The key criterion for classification of the chondriokinesis types was the arrangement of cell organelles during two meiosis phases: metaphase I and telophase I. The autonomous organelles participate in cytoplasmic inheritance; therefore, their precise distribution to daughter cells determines formation of identical viable microspores. In this study, the course of chondriokinesis during the development of the male gametophyte in *Tinantia erecta* was analyzed. The study was conducted using optical and transmission electron microscopes. During microsporogenesis in *T. erecta*, autonomous cell organelles moved in a manner defined as a neutral-equatorial type of chondriokinesis. Therefore, metaphase I plastids and mitochondria were evenly dispersed around the metaphase plate and formed an equatorial plate between the daughter nuclei in early telophase I. Changes in the ultrastructure of plastids and mitochondria during pollen microsporogenesis were also observed.

## Introduction

In flowering plants (Angiospermae), the male gametophyte is formed in the microsporangia of the anther in two consecutive stages of development: microsporogenesis and microgametogenesis.

During microsporogenesis, the division of the cell nucleus (karyokinesis) takes place in two stages. The first stage is the reduction division of chromosomes and the second stage is the mitotic division (John 1990). During meiosis, in addition to karyokinesis, there are also important processes like chondriokinesis—movement and separation of organelles to the daughter cell and cytokinesis—division of the cytoplasm. Chondriokinesis was observed at the end of the nineteenth century and it was systematized in 1938 by Bąkowski (1938). He distinguished four main types of chondriokinesis in plants: neutral, enveloped, polar, and equatorial. In addition, he described intermediate types, for example neutral-enveloped, and complex types, e.g., neutral-equatorial. The criterion that determined the classification of the chondriokinesis type was the position of organelles in two meiotic phases: metaphase I and telophase I. Currently, the process of chondriokinesis in plants has been supplemented and verified by Tchórzewska (2017).

Plastids and mitochondria are organelles that have their own DNA, the level of which in pollen grains is highly regulated during microgametogenesis (Matsushima et al. 2011; Wang et al. 2010). The modes of inheritance of organellar DNA are maternal, biparental, or paternal (Hansen et al. 2007; Hu 1997; Mogensen 1996). Research on the inheritance of autonomous organelles has shown that about 80% of angiosperms exhibit maternal inheritance of organelles, while the other 20% are inherited from both parents (Zhang et al. 2003). Asymmetrical distribution or degeneration of plastids during microgametogenesis or fertilization prevents paternal transmission and thus leads to maternal inheritance (Hagemann 2002; Mogensen 1996; Nagata et al. 2000; Rodkiewicz et al. 1986; Saito et al. 2000; Schröder and Oldenburg 1990; Sodmergen et al. 1995). Maternal inheritance of mitochondrial DNA has been demonstrated to be much stricter than that of plastid DNA (Chat et al. 1999; Testolin and Cipriani 1997). The modes of the inheritance of plastids and mitochondria are mainly determined by the pattern of their distribution and transmission during microgametogenesis or fertilization (Schröder and Oldenburg 1990; Sodmergen et al. 2002). Characterization of behaviors of plastids and mitochondria during microgametogenesis helps to understand the cytological mechanism of the modes of inheritance of these organelles.

DNA located in autonomous organelles is responsible for cytoplasmic inheritance, which is why their precise distribution determines the formation of viable microspores. Disorders in the separation of these organelles can cause cytoplasmic male sterility (CMS) (Majewska-Sawka et al. 1993; Chase 2006; Hu et al. 2014).

This study was conducted on a poorly known species belonging to the Commelinaceae family—*Tinantia erecta* (Jacq.) Fenzl. The family Commelinaceae exhibits high diversity of the morphology of flowers and inflorescences, which differ even in closely related genera. Furthermore, the family has been poorly described in terms of embryology. To the best of the authors’ knowledge, this is the first description of the development of pollen grains in *T. erecta*. This plant was regarded as a potentially invasive species in Europe. It is said to have escaped from botanical gardens in Portugal and poses a threat to native species (de Almeida and Freitas 2006). Therefore, studies related to the sexual reproduction of this species is very important in relation to the control of its further potential spread.

During microsporogenesis in meiotic cells, in addition to karyokinesis and cytokinesis, autonomic organelles (plastids and mitochondria) are displaced and separated into the daughter cell. The literature on chondriokinesis and cytoplasmic inheritance of plastids and mitochondria is not very extensive; therefore, special attention in this work was paid to this aspect of the male gametophyte development. In addition, changes in the structure of autonomous cell organelles were highlighted.

## Materials and methods

The plants were grown in a greenhouse at 23 °C on a universal slightly acid soil with pH of 5.5–6.5, under a natural photoperiod, depending on the season. This species occurs naturally in Central and South America, where it grows on roadsides and farmlands. *T. erecta* seeds were obtained from the UMCS Botanical Garden (Poland), where the species was deposited after being brought from the Botanical Garden of the Technical University of Dresden (Germany)—catalog number MX-0-DR-003782.

For embryological studies, whole flower buds were used, and their single anthers were collected at various stages of development.

### Preparation for light microscopy and transmission electron microscopy

For histochemical studies, anthers were collected and crushed preparations stained with 1% acetocarmine solution were prepared for chromosome staining (Gerlach 1972) and Lugol’s liquid for starch detection (Baker and Baker 1979). The preparations were closed in a drop of glycerol and analyzed using a Nikon Eclipse *Ni*-U light microscope (Tokyo, Japan). The documentation was made using a Nikon digital camera and NIS-Elements BP software.

For anatomical studies, isolated anthers were fixed in a mixture of 2.5% paraformaldehyde and 2.5% glutaraldehyde in cacodylate buffer (pH 7.2) for 24 h at room temperature. The material was then rinsed in cacodylate buffer and placed in a 2% solution of osmium tetraoxide in deionized water. The material was then dehydrated in a series of alcohols, placed in ethanol, and embedded in an LR White acrylic resin (Sigma). The material was cut into semi-thin sections (1 μm) using a Leica EM UC7 microtome (Wetzlar, Germany) and stained with a 1% toluidine blue solution. The preparations were observed using a Nikon Eclipse *Ni*-U microscope. The material to be observed in the transmission electron microscopy (TEM) was cut into ultrathin sections (60–70 nm) using the Leica EM UC7 ultramicrotome, and then collected into a copper mesh coated with a formvar. The preparations were then stained with 2% uranyl acetate and Reynolds reagent and viewed under a TEM Zeiss EM900 electron microscope operating at 80 kV acceleration voltage (Carl Zeiss AG, Oberkochen, Germany) and equipped with a digital camera with corresponding software ImageSP v. 1.1.2.5.

### Terminology

The terminology used to describe the different exine layers of *T. erecta* is defined according to Hesse et al. (2009).

## Results

**Fig. 1:**
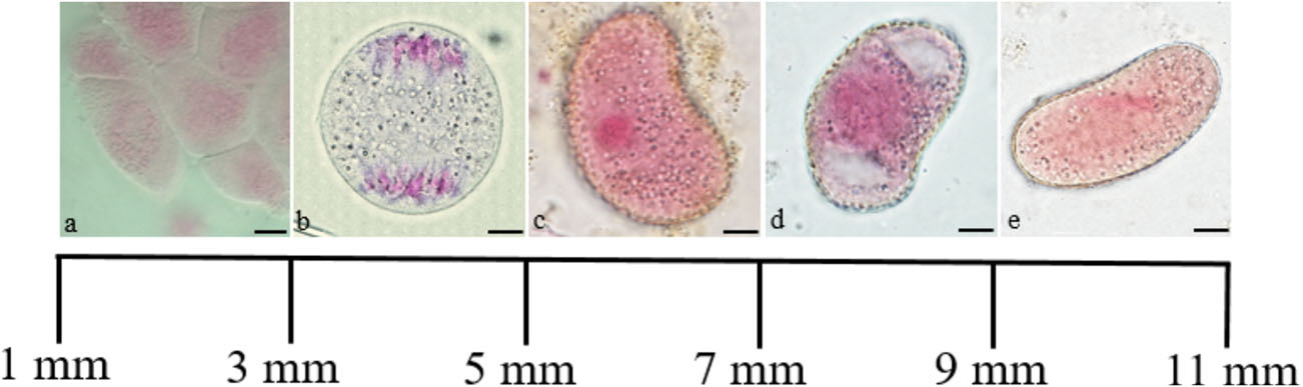
Relationship between the length of the flower bud and the stage of development of *T. erecta* male gametophyte observed in light microscopy (LM) on crushed preparations stained with acetocarmine. **a** Sporogenous tissue. **b** Anaphase I. **c** Free microspore. **d** Vacuolated microspore. **e** Binucleate pollen grain. Scale bars: 10 μm

During the study, correlations were found between the length of the flower bud and the specific stage of meiotic cell development. In the smallest 1–3-mm-long flower buds, sporogenic tissue was present. In flower buds whose length ranged from 3 to 5 mm, successive stages of the meiotic division of microsporocytes to the formation of microspore tetrads were observed. The free microspore stage was observed in buds with a length of 5 to 7 mm. In 7- to 9-mm-long flower buds, the stage of vacuolated microspores was observed. Mature pollen grains in closed anthers were found in 9–11-mm-long flower buds (Fig. 1). Pollen sacs in larger flower buds were opened and pollen was discharged during anthesis.

### Behavior of plastids and mitochondria during microsporogenesis

The microsporogenesis process began with condensation of chromatin in sporogenic tissue cells.

**Fig. 2:**
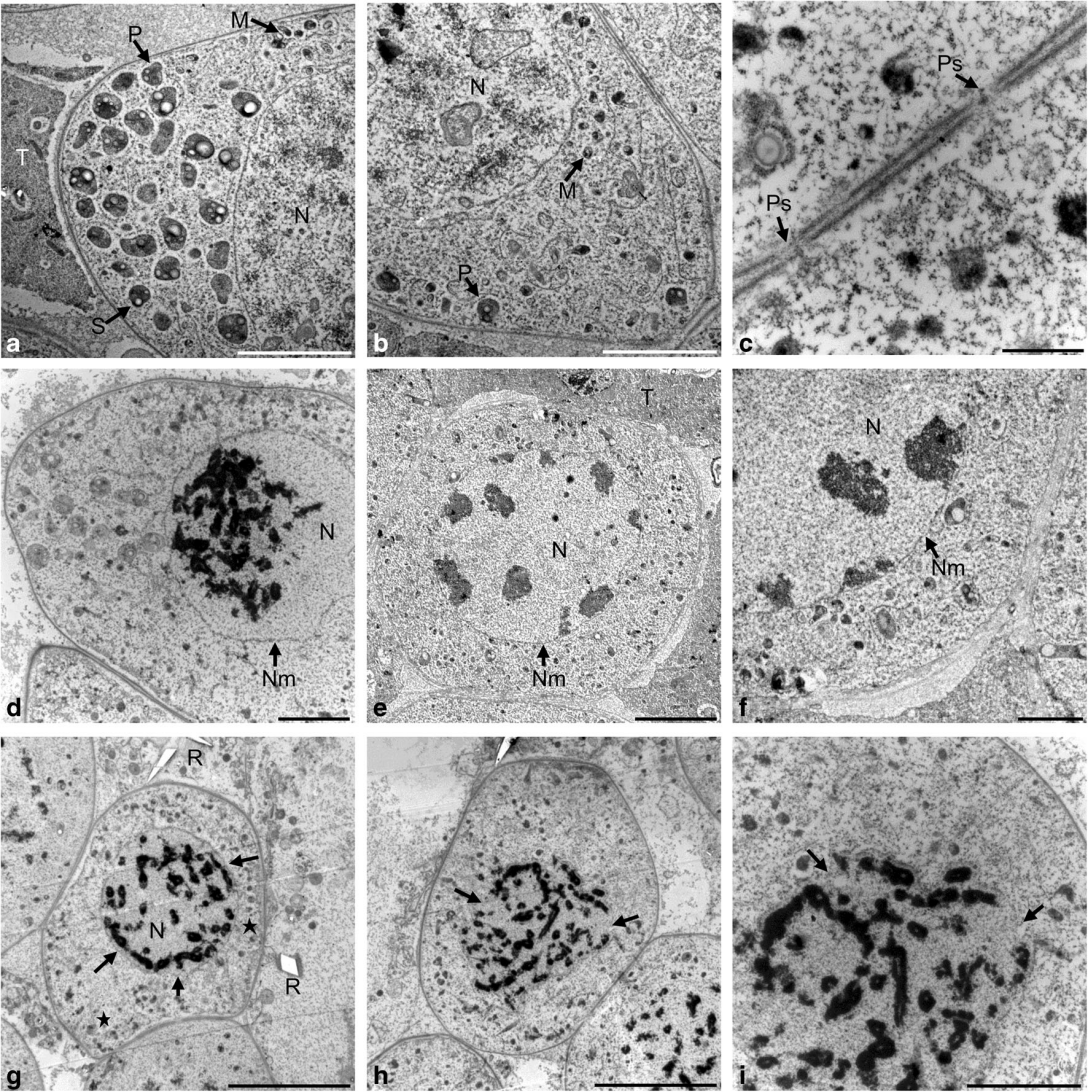
Meiotic prophase I observed in TEM. **a** Grouping of plastids on the cell pole. **b** Distribution of organelles at the opposite pole of the microsporocyte. **c** Plasmodesmata between microsporocytes. **d** Concentration of chromatin in the bouquet stage in zygotene. **e** Pachytene. **f** Clusters of heterochromatin adjacent to the nuclear envelope. **g** Diplotene. **h** Diakinesis. **i** Disintegration of the nuclear membrane at the diakinese stage. Scale bars: 5 μm in **a**, **b**, **e**, **g**, **h**; 2 μm in **d**, **f**, **i**; 500 nm in **c** (*M* mitochondrion, *N* nucleus, *Nm* nuclear membrane, *P* plastid, *Ps* plasdodesmata, *R* raphide, *S* starch grain, *T* tapetum, *Tn* tapetal nucleus)

Microsporocytes in early prophase I had a large centrally located nucleus with loosely packed chromatin. Mitochondria were evenly distributed in the cytoplasm, while plastids formed a cluster at one of the poles of the cell. The plastids had an irregular shape and average dimensions of 410 nm by 250 nm. The mitochondria were small and spherical shape with an average size of 220 to 60 nm (Fig. 2a, b). There were numerous plasmodesmata, i.e., intercellular connections between various microsporocytes through which the endoplasmic reticulum was transported (arrow) (Fig. 2c). During the zygotene, the bouquet stage was observed—condensation and polarization of chromatin on one side of the cell nucleus (Fig. 2d). At the pachytene stage, the chromatin was further condensed and adhered to the nuclear envelope (Fig. 2e). Both in the zygotene and in the pachytene, the cellular organelles were evenly dispersed in the cytoplasm (Fig. 2d–f). In diplotene, condensation of bivalents was observed, which gradually detached from the nuclear envelope (arrow). The plastids and mitochondria were dispersed in the peripheral part of the cytoplasm (star) (Fig. 2g). In the diakinesis phase, the bivalents were completely detached from degrading nuclear envelope (arrow) (Fig. 2h, i), while the plastids and mitochondria moved to the central part of the cell (Fig. 2h).

**Fig. 3:**
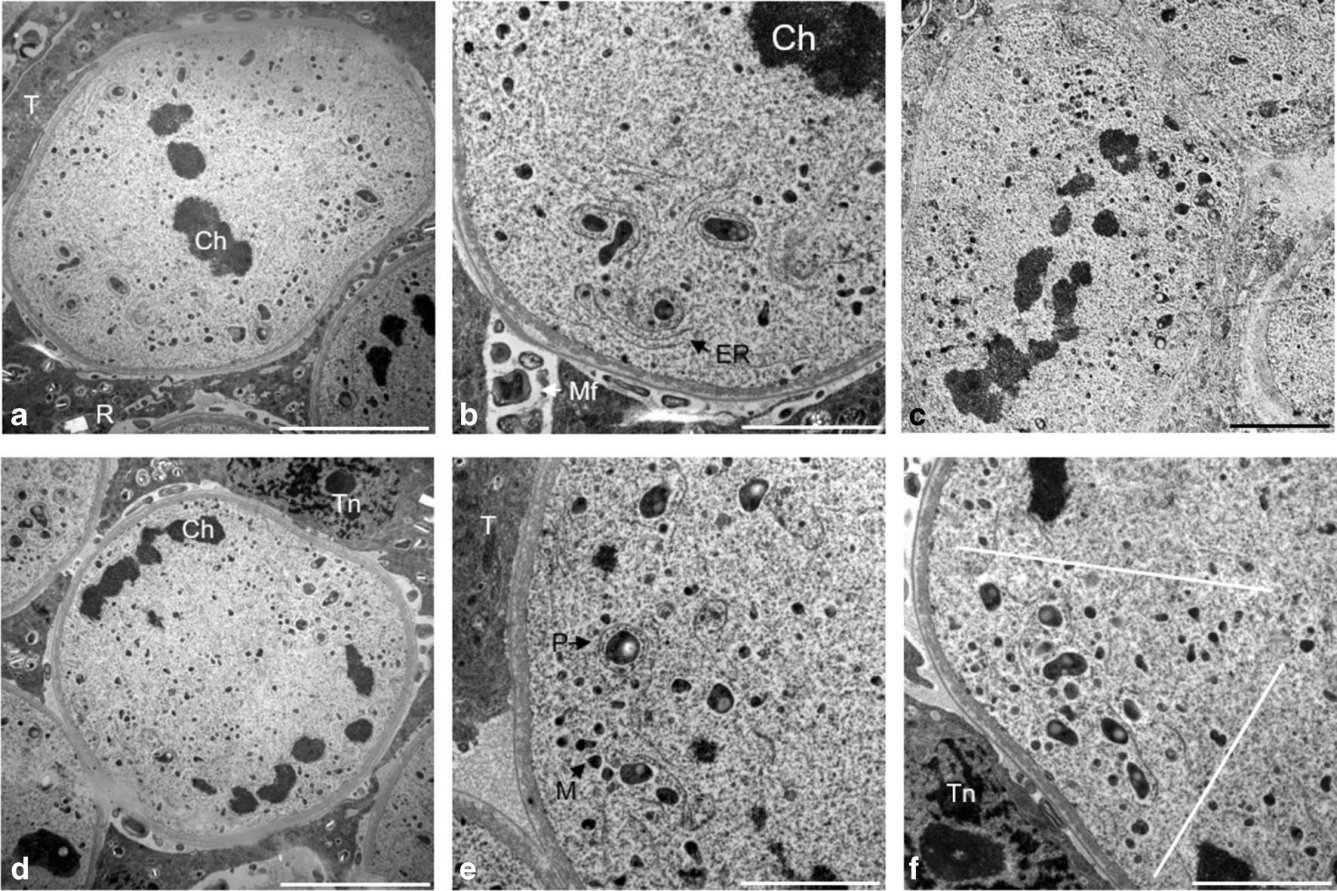
Metaphase I and anaphase I in meiotic cells observed in TEM. **a** Metaphase I. **b** Cell organelles surrounded by the endoplasmic reticulum network. **c** Early anaphase I. **d** Anaphase I. **e** Cell organelles arranged parallel to the wall of the microsporocyte. **f** Organelles grouped in a triangle. Scale bars: 5 μm in **a**, **c**, **d**; 2 μm in **b**, **e**, **f** (*Ch* chromosomes, *M* mitochondrion, *Mf* multimembranous formations, *P* plastid, *R* raphide, *ER* endoplasmic reticulum, *T* tapetum, *Tn* tapetal nucleus)

During the metaphase I, the chromosomes were arranged in the equatorial plate of the division spindle. The cellular organelles were evenly dispersed in the cytoplasm. The microsporocytes were not connected through plasmodesmata, and a thin callose wall surrounding the individual cells was observed. Both in metaphase I and during the entire meiotic division, the cellular organelles were surrounded by an endoplasmic reticulum network (Fig. 3a, b). During early anaphase I when chromosomal bivalents were dispersed to opposite poles of the cell, organelles migrated to the equatorial plate of the cell (Fig. 3c). In late anaphase I, homologous chromosomes were located on the opposite poles of the cell (Fig. 3d). Initially, plastids and mitochondria were arranged in the equatorial zone of the cell and then formed a triangle and moved into the free space of the equatorial plane, which emerged as a result of the dispersal of the chromosomes (Fig. 3e, f).

**Fig. 4:**
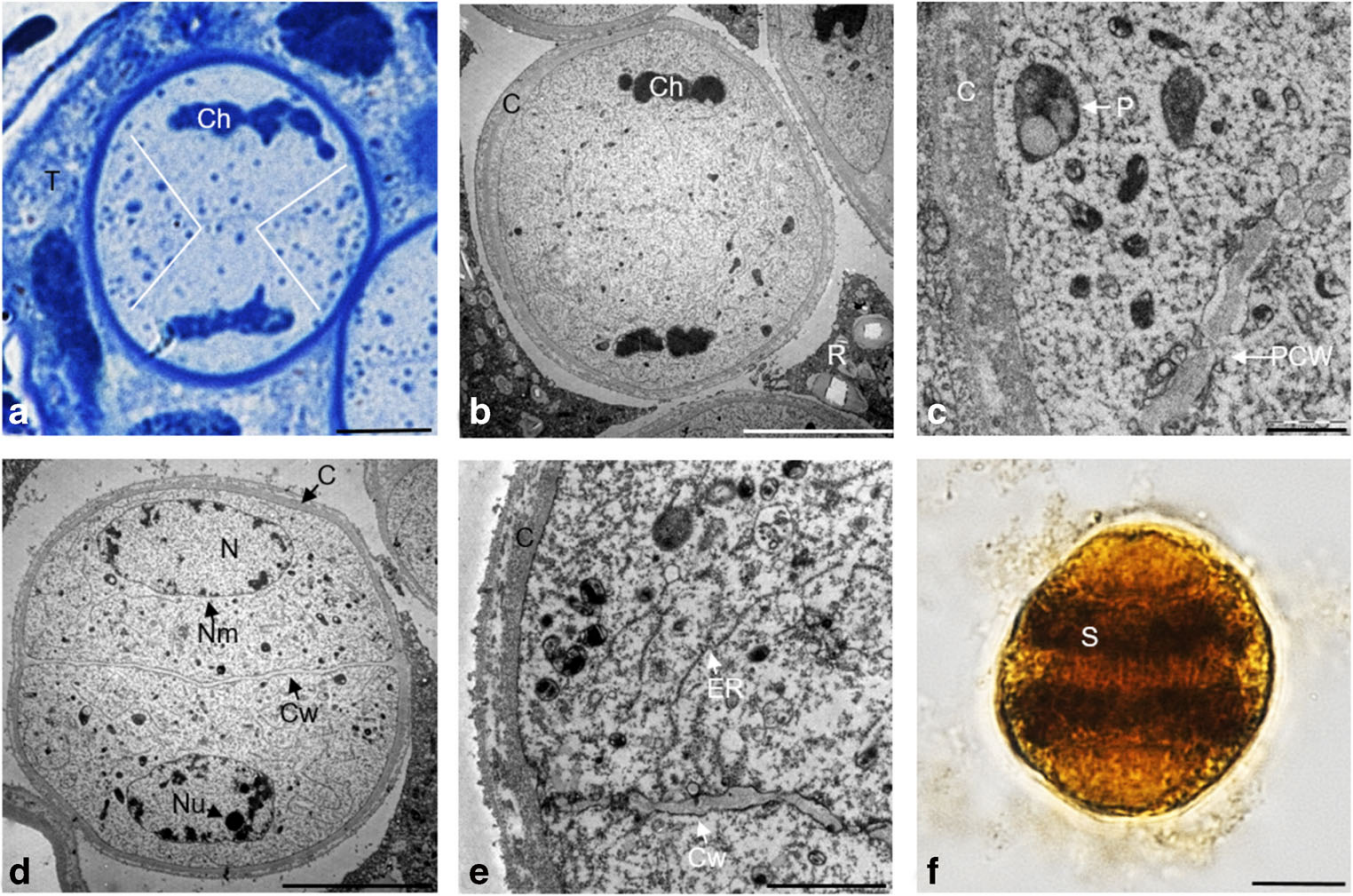
Telophase I and prophase II in meiotic cells. **a** Organelles grouped in triangles during telophase I, semi-thin section stained with toluidine blue. **b** Telophase I, TEM. **c** Vesicles forming the primary cell wall, TEM. **d** Prophase II, TEM. **e** Formed primary cell wall, TEM. **f** Clusters of starch in individual dyad cells, crushed preparation stained in IKI reaction. Scale bars: 10 μm in **a**, **f**; 5 μm in **b**, **d**; 1 μm in **c**, **e** (*C* callose, *Ch* chromosomes, *Cw* cell wall, *M* mitochondrion, *N* nucleus, *Nm* nuclear membrane, *Nu* nucleolus, *P* plastid, *PCW* primary cell wall, *R* raphides, *ER* endoplasmic reticulum, *S* starch grain, *T* tapetum)

In early telophase I, chromatin, which was present on the opposite poles of the dividing cell, was gradually decondensed. The cellular organelles formed two triangular cell aggregates located in the equatorial part of the cell. Dividing meiotic cells were surrounded by a thick layer of callose (Fig. 4a, b). Numerous vesicular structures of different sizes observed in the equatorial plate of the cell formed the primary cell wall (Fig. 4c). In prophase II, daughter nuclei formed, chromatin decondensed, nucleoli appeared, and a nuclear envelope formed around the nuclei (Fig. 4d). In place of the primary cell wall, a callose wall separating the dyad cells formed (Fig. 4e). The cells in prophase II observed on the crushed JKJ-stained preparations had significant amounts of starch that was evenly distributed in the dyad cells (Fig. 4f).

**Fig. 5:**
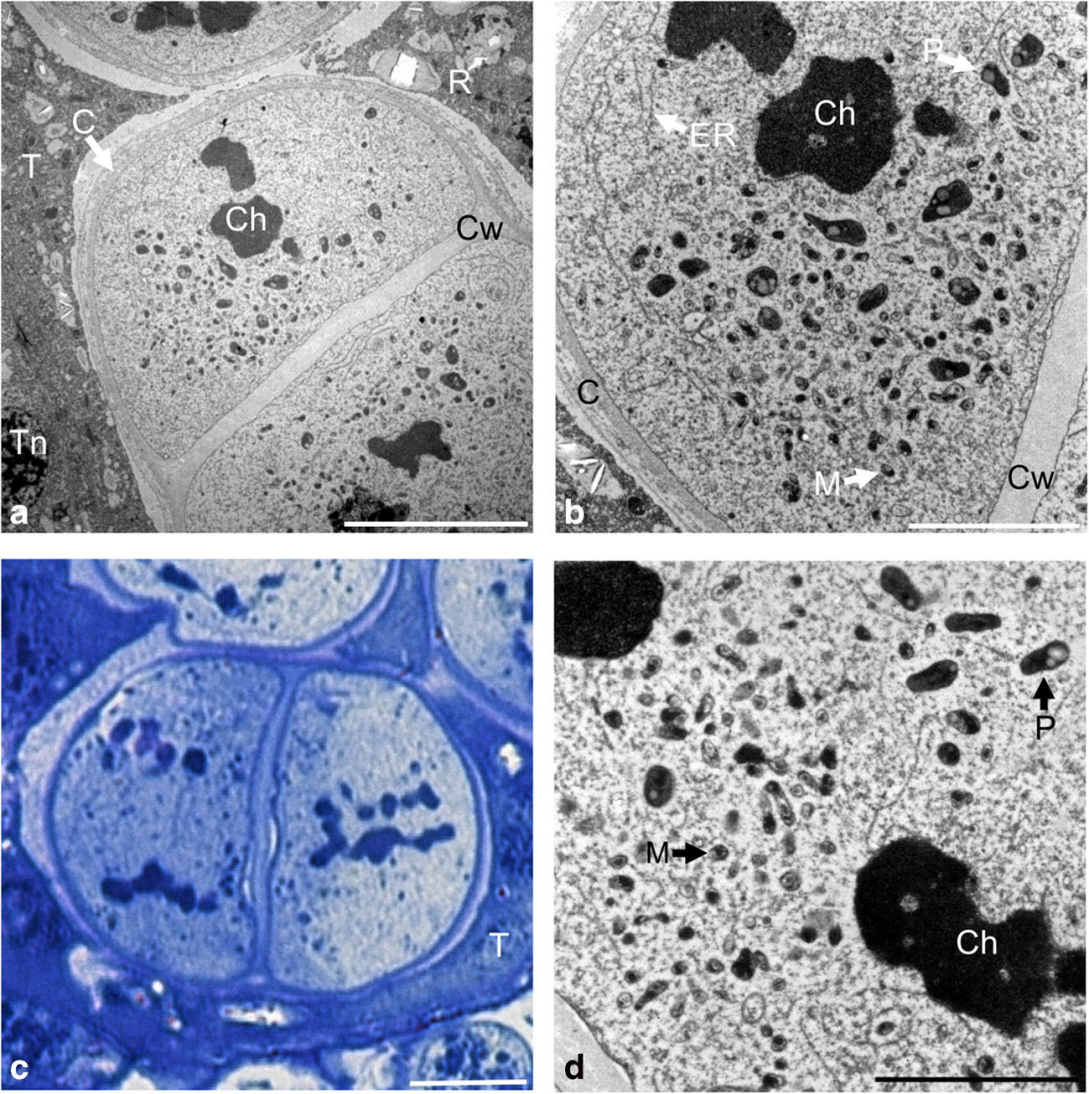
Metaphase II and anaphase II in microsporocytes. **a** Parietal arrangement of cell organelles during metaphase II, TEM. **b** Organelle aggregate, TEM. **c** Early anaphase II, semi-thin preparation stained with toluidine blue. **d** Anaphase II, TEM. Scale bars: 5 μm in **a**; 2 μm in **b**, **d**; 10 μm in **c** (*C* callose, *Ch* chromosomes, *Cw* cell wall, *M* mitochondrion, *P* plastid, *R* raphide, *ER* endoplasmic reticulum, *T* tapetum, *Tn* tapetal nucleus)

During metaphase II, the dyad cells were separated by a thick cell wall at which cellular organelles were grouped. These aggregates were formed by numerous mitochondria and plastids (Fig. 5a, b). During early anaphase II, the organelles were still grouped at the cell wall (Fig. 5c). In late anaphase II, when the sister chromatids were located on the opposite poles of dyad, the organelles were grouped in the equatorial part of the microsporocytes (Fig. 5d).

**Fig. 6:**
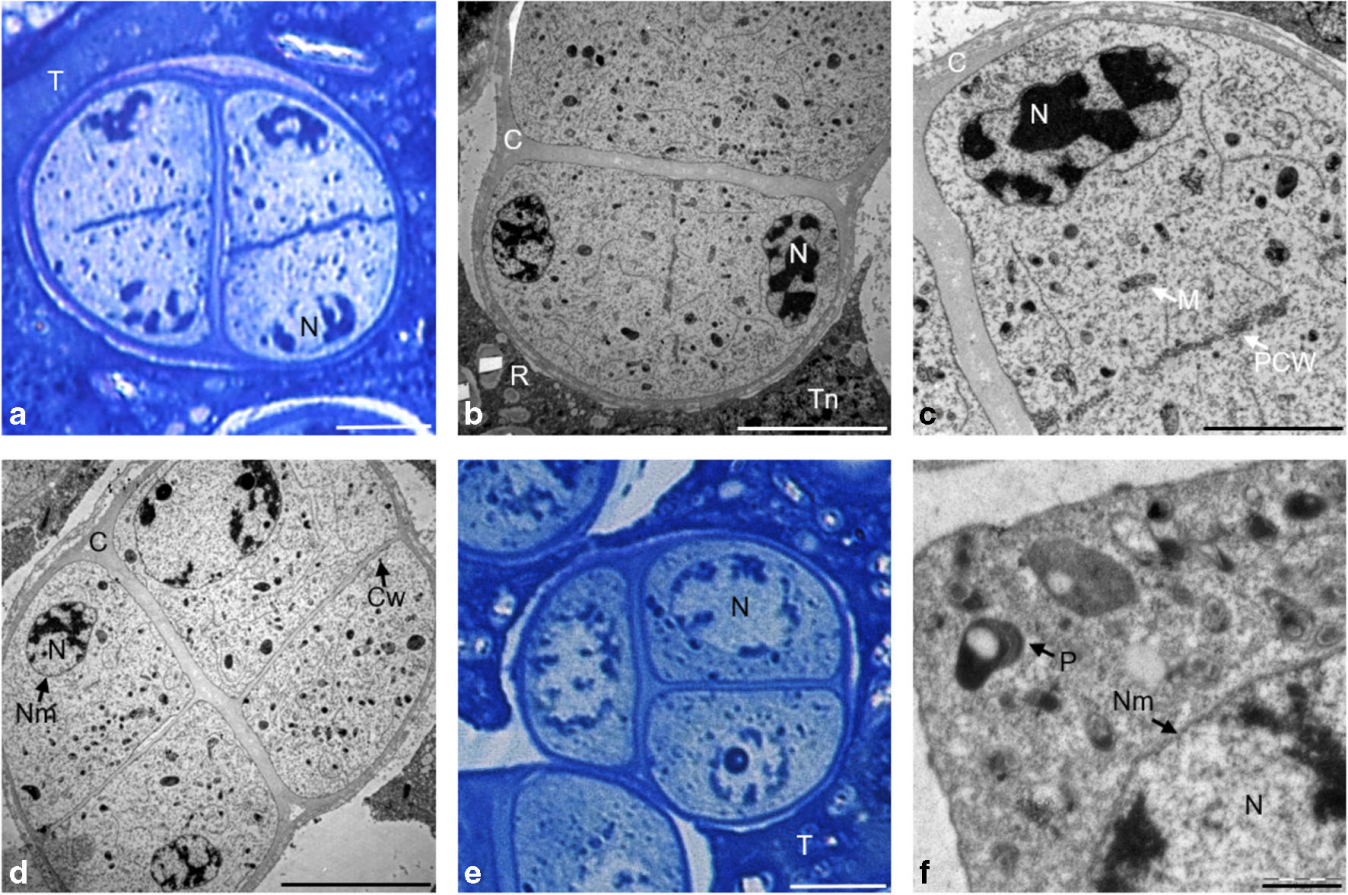
Telophase II and tetrad stage in microspores. **a** Primary wall at the stage of telophase II, semi-thin preparation stained with toluidine blue. **b**, **c** Telophase II, TEM. **d** Tetrahedral arrangement of the microspores in the tetrad, TEM. **e** Alternating arrangement of the microspores in the tetrad, semi-thin preparation stained with toluidine blue. **f** Single microspore in the tetrad, TEM. Scale bars: 10 μm in **a**, **e**; 5 μm in **b**, **d**; 2 μm in **c**; 1 μm in **f** (*C* callose, *Cw* cell wall, *M* mitochondrion, *N* nucleus, *Nm* nuclear membrane, *Nu* nucleolus, *P* plastid, *PCW* primary cell wall, *R* raphide, *T* tapetum)

In microsporocytes during telophase II, cytokinesis occurred after the formation of the four daughter nuclei (Fig. 6a). The centrifugally formed primary cell wall was composed of numerous small vesicles (Fig. 6b, c). The cellular organelles were located in the space between the arising cell wall and the emerging nuclei (Fig. 6c). The microspore tetrad was surrounded by a common callose wall, which degraded over time. The tetrad microspores were arranged in various ways, but most often exhibited tetrahedral and alternate arrangement (Fig. 6d, e). Each of the microspores in the tetrad had mitochondria and plastids that were evenly distributed in the cytoplasm (Fig. 6d–f). After the breakdown of the callose envelope, the anther loculi contained single haploid microspores surrounded by a thin layer of sporoderm.

### Distribution of organelles during microgametogenesis

**Fig. 7:**
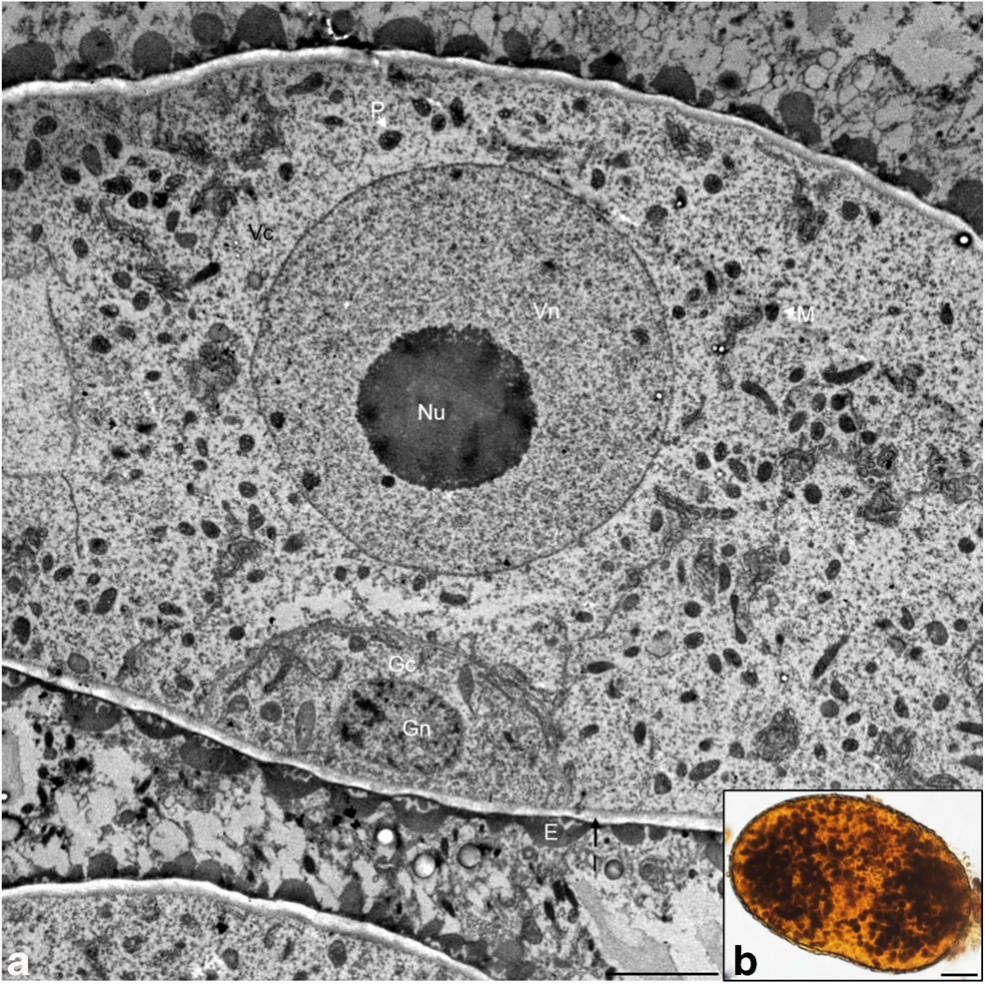
Microgametogenesis stage. **a** Microspore after the first mitotic division, TEM. **b** Starch grains in the microspore, crushed preparation stained in IKI reaction. Scale bars: 1 μm in **a**; 10 μm in **b** (*E* exine, *Gc* generative cell, *Gn* generative nucleus, *I* intine, *M* mitochondrion, *Nu* nucleolus, *P* plastid, *Vc* vegetative cell, *Vn* vegetative nucleus)

The observations showed that the mitotic division of the microspores occurred asymmetrically, resulting in a bicellular pollen grain composed of a larger vegetative cell and a smaller generative cell. The vegetative cell contained a large nucleus with a nucleus as well as numerous cellular organelles located in the cytoplasm, i.e., plastids and mitochondria, endoplasmic reticulum, Golgi vesicles, and numerous free ribosomes. The generative cell was lenticular in shape and was initially collated perietally. The cells were separated from each other by a thin callose wall. A few cellular organelles, an endoplasmic reticulum network, and free ribosomes were visible in the cytoplasm of the generative cell. The binucleate pollen grain was surrounded by a sporoderm with a visible intine layer and an electron-dense layer of exine (Fig. 7a). The JKJ staining showed numerous plastids in the binucleate pollen grain in which the starch granular reserve was stored (Fig. 7b).

**Fig. 8:**
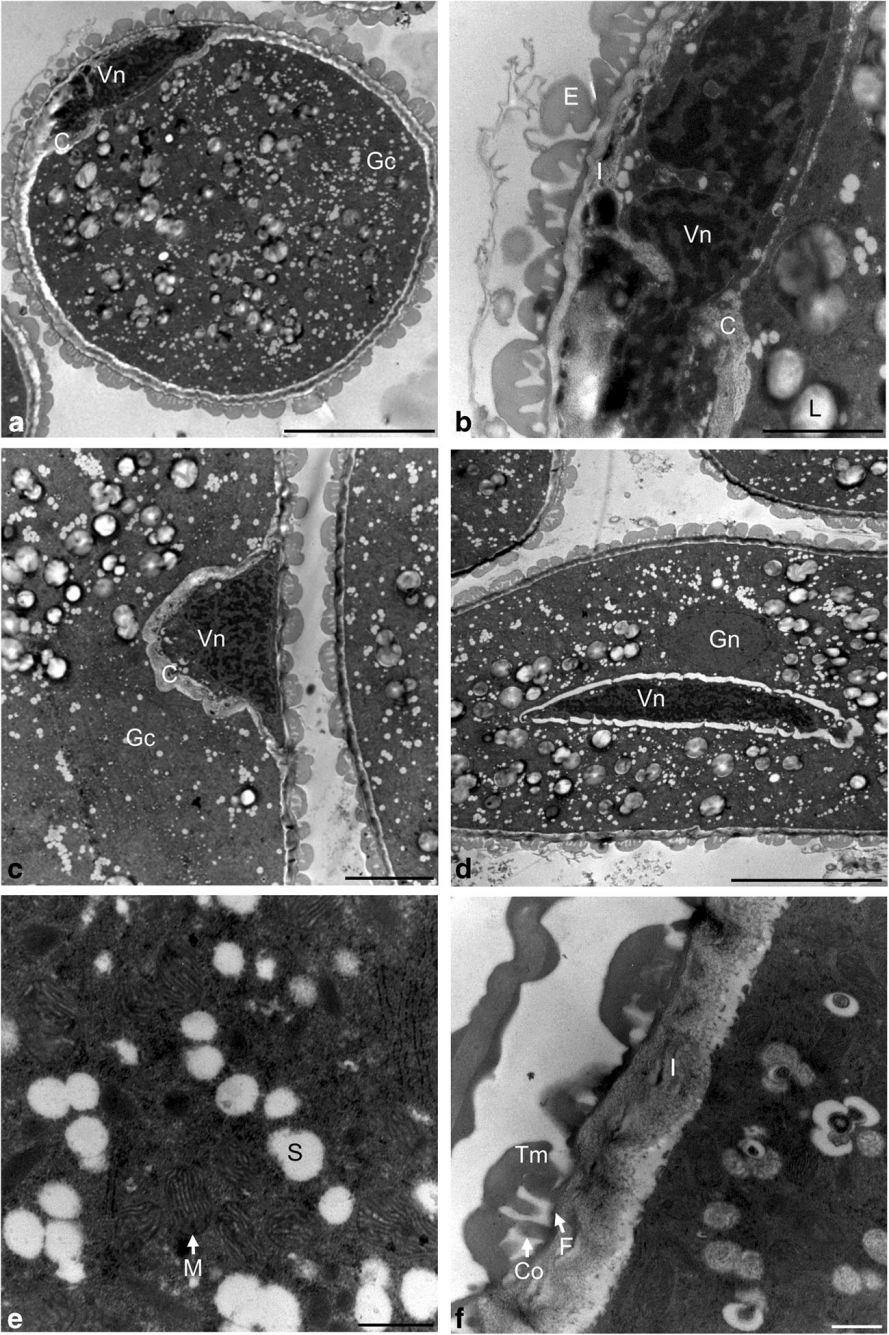
Pollen grain observed in TEM. **a** Parietal position of the generative nucleus. **b** Nucleus of the generative cell. **c** Generative cell detaching from the sporoderm. **d** Central arrangement of the vegetative and generative nucleus. **e** Ultrastructure of the cytoplasm of the pollen grain. **f** Ultrastructure of the pollen grain sporoderm. Scale bars: 5 μm in **a**, **d**; 1 μm in **b**; 2 μm in **c**; 250 nm in **e**, **f** (*C* callose, *Co* columella, *E* exine, F *foot layer*, *Gc* generative cell, *Gn* generative nucleus, *I* intine, *L* lipid body, *M* mitochondrion, *P* plastid, *Vc* vegetative cell, *Vn* vegetative nucleus, *Tm* tectum)

The generative cell was initially located at the wall. Its nucleus occupied a significant portion of the protoplast and had an irregular shape, and the chromatin exhibited a very high degree of condensation (Fig. 8a, b). In the next stage, the generative cell was detached from the pollen grain sporoderm. During this stage, the generative cell changed its shape from spherical to oval (Fig. 8c). The generative cell detached from the intine and sank into the cytoplasm of the vegetative cell. At the same time, it changed its shape to a more elongated and sharp at the ends. In the mature pollen grain, the generative cell was located near the nucleus of the vegetative cell. In the cytoplasm of the vegetative cell, there were numerous mitochondria with visible cristae as well as numerous small vacuoles and plastids, which were completely filled with starch and had an oval shape with an average diameter of 700 nm. The mitochondria in the mature pollen grain changed their shape, and their dimensions were on average 120 nm by 210 nm (Fig. 8d, e). The sporoderm was composed of a thick layer of exine consisting of endo- and ectoexine and an underlying layer of the intine. The ectoexine consisted of a base layer, columellar layers, and tectum, while the intine layer was characterized by a fibrous structure (Fig. 8f).

## Discussion

### Microsporogenesis

In *T. erecta*, a correlation between the length of the flower bud and the stage of microsporogenesis and microgametogenesis was found. The meiosis in *T. erecta* proceeded correctly, and the meiotic division yielded four haploid microspores arranged in a linear, tetragonal, or T-shaped manner, surrounded by a common callose wall. This arrangement of the microspores in the tetrad is the result of successive division of the cytoplasm (Rodkiewicz et al. 1985; De Storme and Geelen 2013). Since successive cytokinesis occurs in bryophytes, Davis (1966) suggests that it is more primitive than simultaneous cytokinesis.

### Chondriokinesis

In the early prophase microsporocytes of *T. erecta*, a short-lived clustering of the plastids at the cell nucleus was observed, whereas the mitochondria were evenly dispersed in the cell. A similar arrangement of organelles was described in, e.g., *Nymphaea alba* (Rodkiewicz and Duda 1988); however, it included both plastids and mitochondria. During zygotene, the characteristic distribution of chromosomes in the nucleus, the so-called bouquet stage, was observed. This stage also occurs in *Allium sativum* (Winiarczyk 2009) and *Zea mays* (Bass et al. 1997). There are two theories explaining the role of the bouquet stage. One theory emphasizes the importance of telomeres in the process of joining homologous chromosomes, and the other one assumes involvement of telomeres in the movement of chromosomes (Dawe 1998).

During telophase I between daughter nuclei, an equatorial plate arises; later, it is replaced by a primary cell wall formed from callose. It can therefore be assumed that the formation of the wall after the first meiotic division determines the dispersal of cellular organelles in the cytoplasm of meiocytes. The second meiotic division takes place in a bicellular meiocyte.

Given the location of cellular organelles in the two key stages of the meiotic division, i.e., metaphase I and telophase I, it can be concluded that *T. erecta* has a neutral-equatorial type of chondriokinesis. This type of chondriokinesis was also observed in *Larix europea* (Rodkiewicz et al. 1984; Bednara and Rodkiewicz 1988), *Larix decidua* (Rodkiewicz and Duda 1988), *Trandescantia virginica* (Rodkiewicz et al. 1984), and ”Vista Rainbow” orchid (Brown and Lemmon 1991). The neutral-equatorial type of chondriokinesis was also observed in the microsporogenesis with simultaneous cytokinesis in *Chamaedorea karwinskiana* (Suessenguth 1921), *Tropaeolum peregrinum* (Sugiura 1928), *Ribes rubrum* (Geneves 1967, 1971), *Podocarpus macrophylla* (Vasil and Aldrich 1970), *Paeonia tenuifolia* and *Campanula rapanculoides* (Dietrich 1973), *Pteridium aquilinum* (Sheffield and Bell 1979), *Dryopteris borreri* (Sheffield and Bell 1979; Sheffield et al. 1983), *Lycopersicon peruvianum* (Pacini and Juniper 1984), *Datura inoxa*, *Nicotiana tabacum* and *Antirrhinum majus* (Dupuis et al. 1988), *Solanum nigrum* (Bhandari and Sharma 1988), *Polystichum loncitis* (Bednara and Rodkiewicz 1988), “Sabine Queen” orchid (Brown and Lemmon 1991), *Lilium longiflorum* (Dickinson and Heslop-Harrison 1970; Tanaka 1991), *Ophioglossum petiolatum* (Brown and Lemmon 2001a, b), *Psilotum nudum* (Gabarayeva 1985; Lee 1982; Tchórzewska et al. 1996; Tchórzewska and Bednara 2011), *Ginkgo biloba* (Wolniak 1976; Wang et al. 1988; Brown and Lemmon 2005), *Taranna gracilipes* (Vinckier and Smets 2007), *Armoracia rusticana* (Winiarczyk et al. 2007), and *Arabidopsis thaliana* (Brownfield et al. 2015).

The basic role of chondriokinesis during meiosis is to ensure an even distribution of organelles between the tetrad microspores (Senjaninova 1927; Geneves 1967; Wolniak 1976; Dupuis 1978; Brown and Lemmon 1982; Tchórzewska 2017), which guarantees formation of identical, metabolically active microspores. It is believed that this process occurs with the same precision as the separation of chromosomes during karyokinesis (Geneves 1967, 1971).

CMS found in higher plants is determined by disorders in the mitochondrial genome (Holford et al. 1991; Chase 2006; Wang et al. 2006; Budar and Berthomé 2007; Mackenzie 2007). These disturbances lead to the formation of non-viable or non-functional pollen grains (Horn et al. 2014). This relatively frequent phenomenon (described in more than 140 species of flowering plants) is probably related to the size of the mitochondrial genome in the plant cell, which ranges from 200 kb in species of the genus *Brassica* (Lebacq and Vedel 1981; Palmer et al. 1983; Palmer and Herbon 1987) and *Oenothera* (Brennicke 1980) to 2400 kb in *Cucumis melo* (Ward et al. 1981).

Chondriokinesis is extremely important not only because of the precise separation of organelles to daughter cells, but also for the normal course of meiosis. Structures such as the equatorial organelle plate (in telophase I and II) and the capsule (in the capsular chondriokinesis) perform similar functions—they determine the areas in which kariokinesis occurs (Bednara et al. 1986, 1995; Kudlicka and Rodkiewicz 1990; Rodkiewicz et al. 1992; Tchórzewska et al. 1996, 2008; Brownfield et al. 2015). The clusters formed by the organelles prevent fusion of nuclei in telophase I, and then separate kariokinetic spindles in metaphase II and telophase nuclei in telophase II. This hypothesis is confirmed by the fact that in meiosis with successive cytokinesis, cellular organelles also cluster and form an equatorial plate in telophase I, but only until the callose wall is formed at the end of the telophase I. After the formation, the organelles are evenly dispersed in the cytoplasm of both dyad cells. Therefore, it can be concluded that there is a close relationship between the type of chondriokinesis and the type of cytokinesis in meiosis during microsporogenesis (Bednara et al. 1986; Tchórzewska 2017).

Another function that can be attributed to chondriokinesis is related to the role of cellular organelles in determining the polarization of myocytes. An additional role of chondriokinesis is to establish the relationship between plastids and the organization of the tubulin cytoskeleton in a plant cell (Tchórzewska 2017).

### Changes in the ultrastructure of plastids and mitochondria during microsporogenesis

Changes in the ultrastructure of plastids and mitochondria during microsporogenesis and development of *T. erecta* pollen grains were observed using a transmission electron microscope. In early prophase I, the presence of numerous starch grains was found in the microsporocytes. The starch did not undergo degradation, was present during the whole pollen grain development process, and filled the entire stroma in mature pollen grains. In addition, it was noticed that the shape of the plastids changed from irregular to oval. It can be assumed that the observed changes represented differentiation of proplastids into amyloplasts through the accumulation of starch. Similar results were obtained by Maruyama (1968), who studied changes in the ultrastructure of cell organelles during the development of pollen grains in *Tradescantia paludosa*. He found that starch was accumulated in cells starting from diakinesis and that it was not degraded during the further stages of meiotic division. Furthermore in mature pollen grains, the starch formed one or two grains filling the whole stroma. The author also observed a change in the shape from elliptical to ovoid. In *Lilium longiflorum* and *Hyacinthiodes non-scripta*, starch was degraded already in early prophase I and reappeared at the stage of the young pollen grain (Dickinson and Heslop-Harrison 1970, 1977; Luck and Jordan 1980).

A number of ultrastructural changes were also observed in the mitochondria. They changed their shape from spherical to oval and their size increased. In the prophase mitochondria, the matrix was electron-thin and the cristae were poorly developed. The mitochondria in the mature pollen grain had an electron-dense matrix with well visible cristae. In the study of mitochondria during microsporogenesis in *Tradescantia paludosa*, Maruyama (1968) observed their rod-like shape and a few weakly visible cristae in preprophase I and early prophase I. During meiosis, the mitochondria changed their shape to spherical, while they gradually lost their internal structures. During the mitotic division, the mitochondria had an elongated shape and very well visible cristae. Similar results were obtained by Luck and Jordan (1980), who investigated microsporogenesis in *H. non-scripta*; they observed that the mitochondria were large with an elongated or spherical shape only in meiotic interphase and leptotene. The authors believe that changes in the shape and number of mitochondria observed in situ are associated with cellular respiration, phosphorylation, or other metabolic processes occurring in the tapetum and in the meiotically dividing cells (Lehninger 1965; Warmke and Lee 1978; Lee and Warmke 1979; Luck and Jordan 1980).

### Cytoplasmic inheritance of plastids and mitochondria during microgametogenesis

Autonomous cellular organelles, i.e., plastids and mitochondria, are the only structures in the cell with their own DNA and, therefore, they participate in the cytoplasmic inheritance. The pattern of plastid inheritance is mainly determined by the pattern of distribution and transmission of plastids during microgametogenesis or fertilization (Schröder and Oldenburg 1990). In angiosperms, both plastids and mitochondria can be inherited from the mother, father, or both parents. Studies on the inheritance of plastids have shown maternal inheritance of organelles in about 80% of angiosperms; in the other cases, plastids are probably inherited from both parents. The maternal inheritance of organelles is controlled by several different cytological mechanisms. These include physical exclusion of organelles during PMI, elimination of organelles by formation of enucleated cytoplasmic bodies (ECB), autophagic degradation of organelles during the development of the male gametophyte, digestion of organelles after fertilization, and digestion of organelle DNA in the generative cell shortly after PMI (Sears 1980; Hagemann and Schröder 1989; Mogensen 1996; Logan 2006; Nagata 2010).

Based on the behavior of plastids, Hagemann and Schröder (1989) have proposed four cytological mechanisms for maternal or biparental plastid inheritance and classified them into four types: (1) Lycopersicon type, where microspore plastids are not transmitted into the generative cell during microspore mitosis; (2) Solanum type, where the newly formed generative cell contains plastids that degenerate before fertilization; (3) Triticum type, where both the generative cell and the sperm cell contain plastids. However, during the process of fertilization, the plastids are stripped off the sperm nucleus and are therefore not transmitted to the zygote. (4) Pelargonium type, where the generative cell and the sperm cell that is destined to fertilize the egg cell contain plastids, which are regularly transmitted into the zygote. The first three types lead to maternal inheritance of plastids, whereas the last one leads to biparental inheritance of plastids.

The result of present study shows that plastids in *T. erecta* are transmitted to the cytoplasm of the generative cell, which suggests biparental inheritance of plastids. However, classification of the inheritance type is impossible at this point because plastids can be excluded from the generative cell during fertilization.

Several mechanisms of maternal mitochondrial inheritance have been described: reduction or elimination of mitochondria in a sperm cell fertilizing an oocyte, prevention of the entry of a sperm cell cytoplasm into an egg cell, or degradation of male mtDNA (Russell 1985; Miyamura et al. 1987; Sodmergen et al. 1992; Mogensen 1996).

The elimination of mitochondria in the sperm cell can occur during cell maturation before the fertilization process (Mogensen and Rusche 1985). The mechanism of elimination consists in the transport of mitochondria into concavities in the sperm cell membrane followed by expulsion of the mitochondria to the cytoplasm of the vegetative cell. This process results in reduction of the quantity of cytoplasm and cytoplasmic organelles surrounding the sperm nucleus. It seems that this phenomenon may be relatively common in the process of maturation of sperm cells (Mogensen 1996).

In this study, we observed the presence of mitochondria in the generative cell of *T. erecta* during microgametogenesis. As in the case of plastids, the classification of mitochondria inheritance suggests biparental inheritance. Plant mitochondria and plastids are typically inherited maternally. In some cases, plastids and mitochondria can be inherited in different ways, for example, *Musa acuminata* shows paternal inheritance of mitochondria with maternal inheritance of plastids, whereas mitochondria in cucumber are inherited paternally, while plastids are inherited maternally (Fauré et al. 1994; Havey 1997). An accurate classification of the type of cytoplasmic inheritance of plastids and mitochondria requires further investigation.

### Microgametogenesis

In free microspores, mitotic division starting microgametogenesis occurred. In Batygina’s (1981) research, it was shown that the resulting generative cells in many Commelinaceae species have different shapes. In *Pyrrhemia fuscata* and *Rheo discolor*, the generative cell is initially round or ovoid, and is elongated or sickle-shaped during maturation. The generative cell is ovoid-round in *Aeilema spiratum*, spindle-shaped in *Murdannia simplex*, or spiral in *Commelina forskalaei*. In contrast, the nuclei of vegetative cells of all species are round and large.

During detachment from the pollen grain sporoderm, the generative cell of *T. erecta* changed its shape from oval to spindle-shaped. It is believed that the change in the shape of the generative cell is an adaptation to its transport in the growing pollen tube (Sanger and Jackson 1971; Cresti et al. 1984; Bednarska 1988; Raghavan 1997; Tütüncü Konyar 2017). The nucleus of the vegetative cell in *T. erecta* remained round with a large, prominent nucleolus until germination into the pollen tube. It was observed that the degradation of the vegetative nucleus and the mitotic division of the generative nucleus in species belonging to Commelinaceae occur only in the pollen tube. Mitosis results in formation of two sperm cells differing in the shape in many species (Batygina 1981).

## Conclusion

Our work has shown that the distribution of mitochondria and plastids during microsporogenesis and microgametogenesis in *T. erecta* is a dynamic process, and exhibits different configurations. Given the location of autonomous organelles (plastids and mitochondria) in the two key stages of the meiotic division, i.e., metaphase I and telophase I, it can be concluded that *T. erecta* has a neutral-equatorial type of chondriokinesis.
